# Effects of an attention demanding task on dynamic stability during treadmill walking

**DOI:** 10.1186/1743-0003-5-12

**Published:** 2008-04-21

**Authors:** Jonathan B Dingwell, Roland T Robb, Karen L Troy, Mark D Grabiner

**Affiliations:** 1 Department of Kinesiology & Health Education, University of Texas, 1 University Station, Mail Stop D3700, Austin, TX 78712, USA; 2 Department of Movement Sciences, University of Illinois at Chicago, 1919 West Taylor St., Chicago, IL 60612, USA

## Abstract

**Background:**

People exhibit increased difficulty balancing when they perform secondary attention-distracting tasks while walking. However, a previous study by Grabiner and Troy (*J. Neuroengineering Rehabil*., 2005) found that young healthy subjects performing a concurrent Stroop task while walking on a motorized treadmill exhibited *decreased *step width variability. However, measures of variability do not directly quantify how a system responds to perturbations. This study re-analyzed data from Grabiner and Troy 2005 to determine if performing the concurrent Stroop task directly affected the dynamic stability of walking in these same subjects.

**Methods:**

Thirteen healthy volunteers walked on a motorized treadmill at their self-selected constant speed for 10 minutes both while performing the Stroop test and during undisturbed walking. This Stroop test consisted of projecting images of the name of one color, printed in text of a different color, onto a wall and asking subjects to verbally identify the color of the text. Three-dimensional motions of a marker attached to the base of the neck (C5/T1) were recorded. Marker velocities were calculated over 3 equal intervals of 200 sec each in each direction. Mean variability was calculated for each time series as the average standard deviation across all strides. Both "local" and "orbital" dynamic stability were quantified for each time series using previously established methods. These measures directly quantify how quickly small perturbations grow or decay, either continuously in real time (local) or discretely from one cycle to the next (orbital). Differences between Stroop and Control trials were evaluated using a 2-factor repeated measures ANOVA.

**Results:**

Mean variability of trunk movements was significantly reduced during the Stroop tests compared to normal walking. Conversely, local and orbital stability results were mixed: some measures showed slight increases, while others showed slight decreases. In many cases, different subjects responded differently to the Stroop test. While some of our comparisons reached statistical significance, many did not. In general, measures of variability and dynamic stability reflected different properties of walking dynamics, consistent with previous findings.

**Conclusion:**

These findings demonstrate that the decreased movement variability associated with the Stroop task did *not *translate to greater dynamic stability.

## Introduction

Falls pose a significant and extremely costly [1] health care problem for the elderly [2] and patients with gait disabilities [3-5]. One recent meta-analysis found that abnormalities of gait or balance were the most consistent predictors of future falls [6]. Because most falls occur during whole-body movements like walking [7,8], understanding the mechanisms humans use to maintain dynamic stability during walking is critical to addressing this momentous clinical problem effectively [9,10]. The ability to maintain balance during walking can be negatively affected by concomitant information processing and this effect appears to increase with age [11]. These effects can be studied using various dual-task paradigms, which require subjects to perform an attention demanding secondary task while simultaneously performing a primary task like walking. Dual-task paradigms assume humans possess limited information processing capacity. When performing both primary and secondary tasks, each of which require some level of attention, a negative influence on the performance of either task may indicate structural interference or capacity interference [11]. The former is associated with tasks that share common input and output resources whereas the latter is associated with exceeding the total information processing capacity.

Dual-task paradigms have been used to investigate walking in part because of the frequency with which walking is performed concurrently with cognitive tasks. The changes in reaction time and gait-related variables (e.g., [12-15]) reported for older adults during dual-task paradigms have been associated with increased fall-risk. For example, performing a verbal reaction time task during an obstacle avoidance task significantly increases the risk of obstacle contact by young adults [16] and to an even greater extent by older adults [17]. These results broadly suggest that performing cognitive tasks during locomotion may increase the risk of tripping.

The variability of step kinematics has also been strongly linked with falls by older adults. In particular, cross-sectional and prospective studies have consistently linked increased step time variability to falls in the normal aging population [18,19]. Older adults without a history of falls exhibit increased step width and step width variability compared to young adults [20], which likely reflects the increased need for lateral stabilization, despite incurring increased energetic cost [21]. Prospective studies have also shown that increases in stride-to-stride variability of walking speed [22] and/or stride time [19] can discriminate older adults who fall from those who do not.

The apparent relationship between increased fall-risk when performing attention demanding tasks while walking, and the relationship between step kinematic variability and fall risk raises the question of whether attention demanding tasks increase step kinematic variability. Some evidence supports this idea. For example, step time variability of patients with either Parkinson's or Alzheimer's disease is significantly greater than that of healthy controls and also demonstrates *additional *significant increases when performing an attention demanding task while walking [23,24]. Conversely, Grabiner and Troy [25] recently found that young healthy subjects performing a concurrent Stroop task [26] while walking on a motorized treadmill actually exhibited *decreased *step width variability. This Stroop test consisted of projecting images of the name of one color, printed in text of a different color, onto a wall and asking subjects to verbally identify the color of the text. These authors suggested that these changes may have reflected a voluntary gait adaptation toward a more conservative gait pattern that emphasized frontal plane trunk control [25].

While the findings of Grabiner and Troy initially appear counter-intuitive, the biomechanical and physiological significance of changes in gait variability remain an issue of considerable debate. Variability is often assumed to be deleterious, reflecting the presence of unwanted noise in a physiological system.

Alternatively, variability may reflect a desirable trait of an adaptive system that arises from the interaction of multiple control systems [27]. As specifically related to walking, several recent studies found that step width variability can distinguish between healthy young and elderly subjects [28], that step width cannot distinguish between fit and frail elderly adults [29], and that elderly adults with a history of falls may exhibit either too much or too little step width variability [30]. Thus, it remains quite unclear what true clinical implications may be drawn from observed changes in measures of locomotor variability.

One potential reason for this is that statistical measures of variability do not directly quantify how the locomotor system responds to perturbations [10]. Previous work has shown that measures of kinematic variability are not well correlated with measures of dynamic stability that directly quantify the sensitivity of walking kinematics to small perturbations [9,31,32]. Variability may also not be equated with the stability exhibited in response to larger perturbations [33]. The purpose of the present study was therefore to determine how performing a concurrent attention-distracting Stroop task would affect the dynamic stability of walking in young healthy subjects. We analyzed the dynamic stability of upper body kinematics of the subjects tested in same experiments previously reported by Grabiner and Troy [25]. We hypothesized that while these subjects did exhibit decreased step width variability, they would conversely exhibit *increases *in the sensitivity of their upper body (i.e. trunk) movements to the small inherent perturbations that naturally occur during normal walking [9,31,32].

## Methods

Fifteen young healthy individuals (8 male and 7 female, age: 24.5 ± 3.4 years, height: 1.66 ± 0.12 m, and mass: 68.5 ± 8.0 kg) volunteered to participate. The protocol was reviewed and approved institutionally and all subjects provided written informed consent prior to participating. All data were obtained from the same subjects tested during the same experiments previously described by Grabiner and Troy [25]. Data for 2 of these subjects were unusable for the present analyses due to technical difficulties that arose during data collection. Therefore, the results obtained from the remaining 13 subjects are reported here.

Subjects walked on a motorized treadmill at their self-selected constant speed for 10 minutes each, both while walking normally and while concurrently performing an attention demanding Stroop test [26]. During control trials, subjects were asked to walk while looking straight ahead at a wall approximately five meters away. During Stroop test trials, images consisting of the name of one of four colors, printed in text of a different color, were projected onto the wall in letters 15 cm tall. These images changed randomly once every second. The subjects were instructed to verbally identify the color of the text and ignore the word itself. The order of presentation of the Stroop and control conditions was randomly assigned and the entire experiment was performed during a single day.

In addition to the foot marker data used to report step width variability in Grabiner and Troy [25], a retro-reflective marker was also attached to the skin over the 5^th ^cervical/1^st ^thoracic vertebrae (C5/T1) to measure the three-dimensional movements of the upper body during each trial. Our analyses here focused on these upper body movements because over half of the body's mass is located above the pelvis. Thus, maintaining dynamic stability of the trunk is critical for maintaining stability of the body as a whole [9,34,35]. The motions of this C5/T1 marker in the anterior-posterior (AP), mediolateral (ML), and vertical (VT) directions were recorded using an 8-camera motion analysis system (Motion Analysis, Santa Rosa, CA, USA) operating at 60 Hz. Raw marker data were filtered with a zero-lag Butterworth filter with a cutoff frequency of 6 Hz.

The analytical techniques applied also required stationary data [36], but the raw motion data exhibited considerable nonstationarity mainly because subjects "wandered" in the horizontal plane as they walked on the treadmill [9]. To obtain more stationary data, the velocity of each time series (*V*_AP_, *V*_ML_, and *V*_VT_) was calculated using a standard 3-point difference formula [37]:

(1) $$V_{X}\left(n\right)=\frac{D_{X}\left(i+1\right)-D_{X}\left(i-1\right)}{2\Delta t}$$

where *D*_*X*_(*i*) was the displacement in each direction, *X *∈ {*AP*, *ML*, *VT*}, at data sample *i *and Δ*t *= 1/60 sec was the time between data samples. The analysis techniques used here were independent of specific measurement units. Thus, analyzing the dynamical properties of the velocity time series was equivalent to analyzing the dynamical properties of the displacement time series [9,36]. Additionally, each ten-minute time series was first divided into three equal intervals of 200 sec (approximately 150 strides) each to calculate both within- and between-subject variances in each dependent measure. Data for all strides from all trials were analyzed. While the number of strides analyzed was slightly different for each subject and trial, the analyses conducted here were not sensitive to small changes in this parameter [9,38].

**Figure 1:**
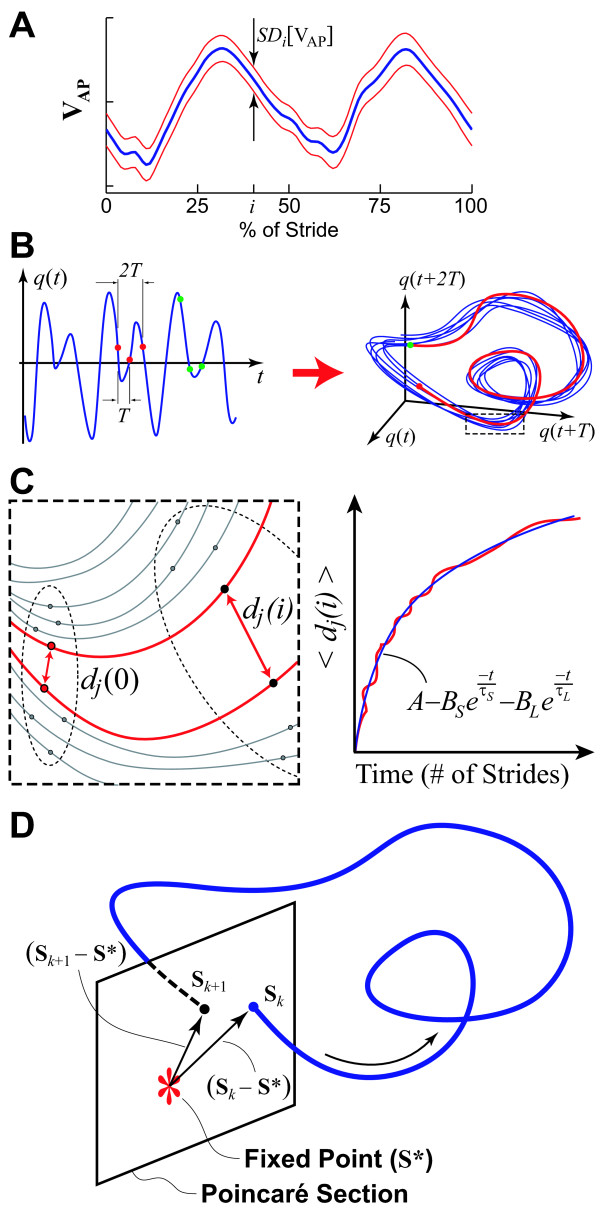
**Schematic representations of dependent measure calculations. *A***: Example of mean ± 1 SD for a typical time series. Between-stride standard deviations are computed at each % of the gait cycle (*i*) and then averaged to compute the MeanSD across the entire gait cycle (Eq. 2). ***B***: An original time series, *q*(*t*), is reconstruction into a 3-dimensional attractor such that **S**(*t*) = [*q*(*t*), *q*(*t*+T), *q*(*t*+2T)]. The two triplets of points indicated in ***A ***and separated by time lags *T *and 2*T *each map onto a single point in the 3D state space. ***C***: Expanded view of a local section of the attractor shown in ***B***. An initial naturally occurring local perturbation, *d*_*j*_(0), diverges across *i *time steps as measured by *d*_*j*_(*i*). The average logarithmic divergence, <*d*_*j*_(*i*)> is computed across all pairs of initially neighboring trajectories and then fit with a double exponential function (Eq. 5). ***D***: Representation of a Poincaré section transecting the state space perpendicular to the system trajectory. The system state at stride *k*, **S**_*k*_, evolves to **S**_*k*+1 _one stride later. The Floquet multipliers quantify whether the distances between these states and the system fixed point, S*, grow or decay across multiple strides (Eq. 8).

To quantify variability, the *V*_AP_, *V*_ML_, and *V*_VT _data for each individual stride were extracted and time-normalized to 101 samples (0% to 100%). Individual strides were differentiated by identifying every other minimum from the vertical movements of the C5/T1 marker [9]. Standard deviations were calculated across all strides at each normalized time increment and then averaged over the normalized stride to produce a single measure of the mean variability ("MeanSD") for each trial (Fig. 1A):

(2) MeanSD(*V*_*X*_) = 〈*SD*_*n *_[*V*_*X*_]〉

where *V*_*X *_denotes the velocity in each direction, *X *∈ {*AP*, *ML*, *VT*}, *n ∈ *{0%, ..., 100%} is an index denoting each percentage of the gait cycle, and 〈·〉 denotes the average over all values of *n *[9].

In theoretical mechanics, stability is defined by how a system's state variables respond to perturbations [39]. For *a*periodic systems that exhibit no discernable periodic structure, "*local stability*" is defined using local divergence exponents [36,40], which quantify how the system's states respond to very small (*i.e*. "local") perturbations continuously *in real time *[9,10,31]. For limit cycle systems, defined as having a constant fixed period, "*orbital stability*" is defined using Floquet multipliers [39] that quantify, discretely *from one cycle to the next*, the tendency of the system's states to return to the periodic limit cycle orbit after small perturbations [32,41,42]. Because human walking is neither strictly periodic, nor strongly aperiodic, both methods were used to assess the sensitivity of walking kinematics to small perturbations during continuous walking.

For both analyses, we first defined appropriate multi-dimensional state spaces for each individual time series using standard delay-reconstruction techniques [9,10,36] (e.g., Fig. 1B):

(3) **S**(*t*) = [*q*(*t*), *q*(*t *+ *T*), *q*(*t *+ 2*T*), ..., *q*(*t *+ (*d*_*E *_- 1)*T*)]

where **S**(*t*) was the *d*_*E*_-dimensional state vector, *q*(*t*) was the original 1-dimensional data [i.e., either *V*_AP_(*t*), *V*_ML_(*t*), or *V*_VT_(*t*)], *T *was the time delay, and *d*_*E *_was the embedding dimension. Time delays were calculated from the first minimum of the Average Mutual Information function [10,36]. An embedding dimension of *d*_*E *_= 5 was used for all data sets, as determined from a Global False Nearest Neighbors analysis [10,36]. Note that these state spaces consisted of the original real-time data (i.e., data were not time normalized).

To quantify *local *stability, the mean local divergence of nearest neighbor trajectories was calculated using a previously published algorithm [40]. For each point **S**(*t*) in state-space, the nearest neighboring point **S**(t*) on an adjacent trajectory was determined (Fig. 1C). For each pair (*j*) of initially nearest neighbors, the subsequent divergence over time between these two points was then calculated:

(4) *d*_*j*_(*i*) = ||**S**(*t *+ *i*Δ*t*) - **S**(t* + *i*Δ*t*)||_2_

where *d*_*j*_(*i*) was the Euclidean distance between the two trajectories after each discrete time step *i *(i.e. *i*Δt seconds). This local divergence was computed out to 10 seconds (*i *= 600 samples) beyond each initial perturbation. This process was repeated for all points from the data set and then averaged to define the *mean *local divergence curve, 〈*d*_*j*_(*i*)〉, where 〈∙〉 denotes the arithmetic mean over all values of *j *(Fig. 1C).

For purely deterministic "chaotic" systems, these mean local divergence curves would be *linear*, reflecting a *constant *exponential rate of divergence [36,40,43], and their slope would approximate the maximum finite-time Lyapunov exponent for the system. Since the curves we obtained (e.g., Fig. 1C and [9,10]) were clearly *not *linear, there was no basis for defining a true Lyapunov exponent for human walking [36,43]. Nevertheless, these local divergence exponents still provided rigorously defined metrics for estimating the sensitivity of human walking to small intrinsic perturbations [10,35]. To parameterize this sensitivity, we instead fit a double-exponential function to each mean divergence curve [35]:

(5) $$\langle d_{j}(i)\rangle \;=A-B_{S}e^{-t/\tau_{S}}-B_{L}e^{-t/\tau_{L}}$$

where *τ*_*S *_and *τ*_*L *_(*τ*_*L *_>> *τ*_*S*_) represent the time constants that describe how quickly 〈*d*_*j*_(*i*)〉 saturates to A, and B_S _and B_L _determine the size of the effect the dynamics at each timescale have on 〈*d*_*j*_(*i*)〉 [35]. Eq. 5 was fit to each divergence curve using the 'fmincon' function in Matlab. This function requires an initial guess of the parameter values and for most of the 234 time series analyzed, the results were not particularly sensitive to this choice. For ~40 time series (~17%), the initial guess had to be adjusted an additional 1–3 times to obtain good curve fits. The exponents *τ*_*S*_^-1 ^and *τ*_*L*_^-1 ^are mathematically directly analogous to the "short-term" and "long-term" local divergence exponents we have used previously [35]. Values of *A*, *B*_*S*_, *τ*_*S*_, *B*_*L*_, and *τ*_*L *_were computed for each trial for each subject for each test condition.

*Orbital *stability was quantified by calculating the Floquet Multipliers (FM) for the system [39] based on well-established techniques [32,41,42,44]. Because Floquet theory assumes the system is strictly periodic, the state space data (Eq. 3) for each stride were first time-normalized to 101 samples (0% to 100%). We could then define a Poincaré map (Fig. 1D) for the system at any chosen % of the gait cycle as:

(6) **S**_*k*+1 _= **F**(**S**_*k*_)

where *k *was an index enumerating the individual strides and **S**_*k *_denoted the system state for the single chosen % of the gait cycle. Limit cycle trajectories correspond to fixed points in each Poincaré map:

(7) S* = **F**(S*)

For our walking data, we chose Poincaré sections at 0%, 25%, 50%, 75%, and 100% of the gait cycle [32,42]. We defined the fixed point at each Poincaré section by the average trajectory across all strides within a trial. Orbital stability at each Poincaré section was estimated by quantifying the effects of small perturbations away from these fixed points, using a linearized approximation of Eq. (6):

(8) [**S**_*k*+1 _- S*] ≈ *J*(S*) [**S**_*k *_- S*]

where *J*(S*) defined the Jacobian matrix for the system at each Poincaré section. Floquet multipliers (FM) are the eigenvalues of *J*(S*) [39,41,44]. Deviations away from the fixed point are multiplied by FM by the subsequent cycle (Fig. 1D). If the magnitude of the largest FM is < 1, these deviations decay and the limit cycle is orbitally stable. Smaller FM imply greater stability. We therefore computed the magnitudes of the maximum FM (MaxFM) for each Poincaré section for each trial for each subject for each test condition.

For each dependent measure computed, differences between control (CO) walking and Stroop test (ST) walking were evaluated using a two-factor (Subject × Condition) repeated measures (i.e., 3 intervals per trial) balanced ANOVA for randomized block design, where Subject was a random factor. For the local dynamic stability variables (*A*, *B*_*S*_, *τ*_*S*_, *B*_*L*_, and *τ*_*L*_), the data were first log transformed to satisfy linearity and normality constraints. For each dependent measure, p-values for each main effect and for Subject × Condition interaction effects were obtained. Finally, linear and quadratic regression analyses were run to determine if differences in movement variability (i.e., MeanSD) across subjects were generally correlated with differences in either local or orbital stability.

## Results

**Figure 2:**
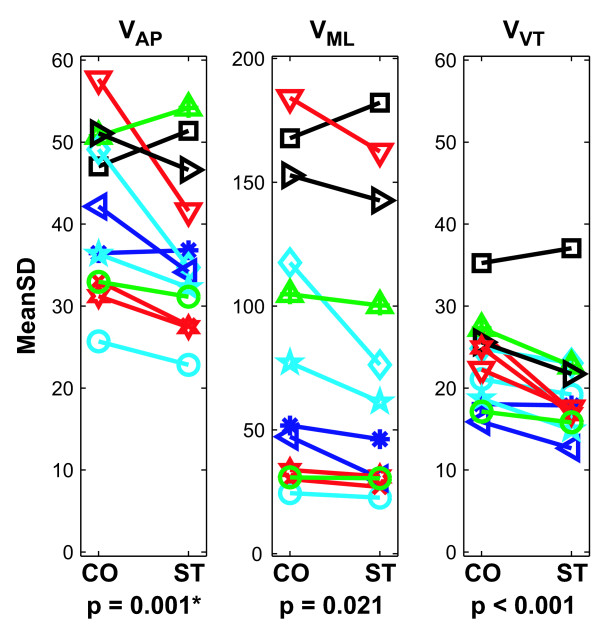
**Kinematic variability (MeanSD) results for trunk velocities in the anterior-posterior (AP), mediolateral (ML), and vertical (VT) directions.** Note that the vertical scale is different for the ML direction compared to the AP and VT directions. Nearly all subjects exhibited greater variability during the Control (CO) walking trials, particularly in the AP and VT directions. Variability of ML movements was much greater than that of AP and VT movements. The "*" indicates a statistically significant Subject × Condition interaction effect (p = 0.008).

The mean variability (MeanSD) of upper body (i.e., trunk) movements was significantly greater during Control (CO) walking than during Stroop (ST) walking (p ≤ 0.021) for all three principle directions: *V*_AP_, *V*_ML_, and *V*_VT _(Fig. 2). As expected, trunk movement variability was greatest in the medio-lateral (ML) direction. Additionally, there was a significant Subject × Condition interaction effect for movements in the anterior-posterior (AP) direction (p = 0.008), indicating that while most subjects' AP variability decreased during the Stroop test, this was not true for all subjects (Fig. 2).

**Figure 3:**
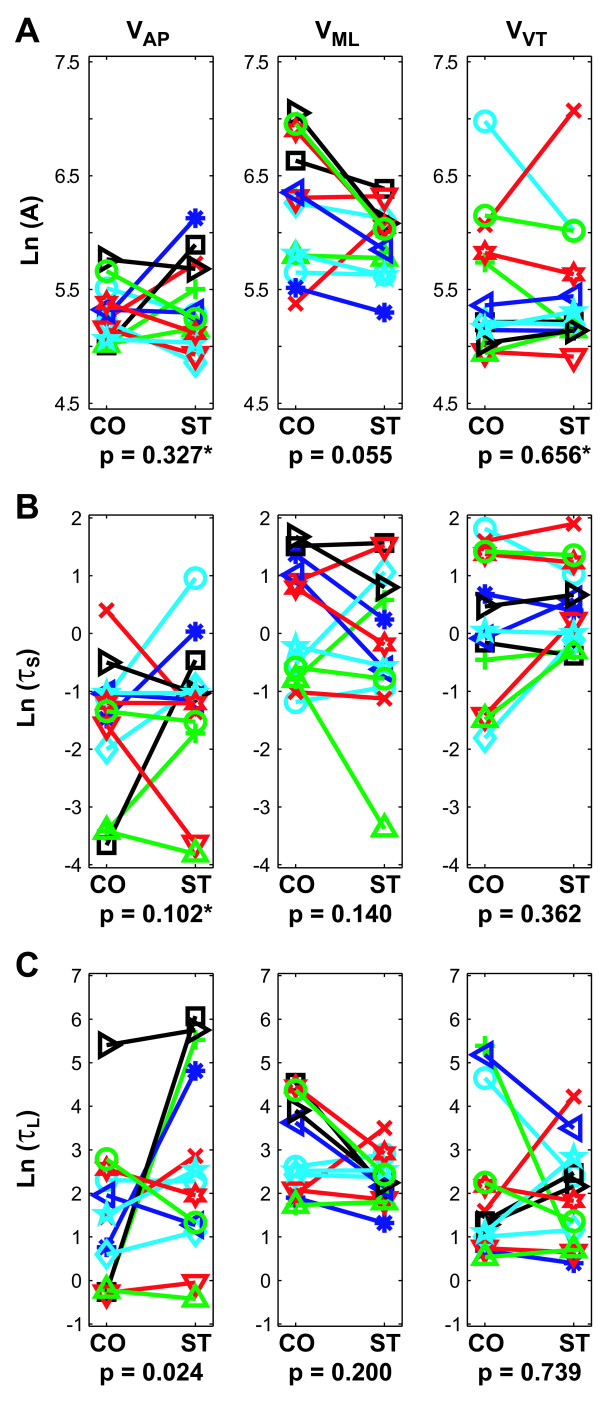
**Local dynamic stability results for AP, ML, and VT trunk velocities.** These data were log transformed to satisfy linearity and normality constraints of the ANOVA analyses. ***A***: Divergence amplitudes (*A *in Eq. 5) were slightly greater in the ML direction (p = 0.055) during Control (CO) walking relative to Stroop test (ST) walking. ***B***: Short-term time constants (*τ*_*S *_in Eq. 5) were not significantly different between the 2 tasks. ***C***: Long-term time constants (*τ*_*L *_in Eq. 5) were significantly smaller (i.e., indicating greater local instability) for the CO walking condition for movements in the AP direction (p = 0.024). This same trend was observed in the ML direction, but was not statistically significant (p = 0.200). The "*" indicate statistically significant Subject × Condition interaction effects (p < 0.05). In general, the Stroop test led to slightly more stable movements in the AP direction, but slightly more *un*stable movements in the ML direction, compared to CO walking.

Overall, the Stroop test led to either no changes or inconsistent changes in local stability. The asymptotic amplitudes of the local divergence curves ('*A*' in Eq. 5; Fig. 3A) tended to be greater for CO walking than for ST walking for ML movements (p = 0.055). For AP and VT movements, the were no significant differences for Condition (p > 0.32), but there were statistically significant Subject × Condition interactions (p = 0.021 and p = 0.036 for AP and VT directions, respectively). Short-term time constants ('*τ*_*S*_' in Eq. 5; Fig. 3B) tended to be slightly larger (i.e., more stable) during ST walking than CO walking for AP movements (p = 0.102). However, the significant Subject × Condition interaction (p = 0.001) indicated that different subjects exhibited different responses. Long-term time constants ('*τ*_*L*_' in Eq. 5; Fig. 3C) were significantly larger (i.e., more stable) during ST walking than CO walking for AP movements (p = 0.024), but were not significantly different for ML (p = 0.200) or VT (p = 0.739) movements. While the Subject × Condition interaction effects were not statistically significant (0.10 < p < 0.30), differences between subjects were evident in the data (Fig. 3C). Short-term and Long-term scaling coefficients ('*B*_*S*_' and '*B*_*L*_' in Eq. 5; data not shown) exhibited no significant differences between the two walking conditions (0.24 < p < 0.67 for *B*_*S *_and 0.15 < p < 0.93 for *B*_*L*_, respectively).

**Figure 4:**
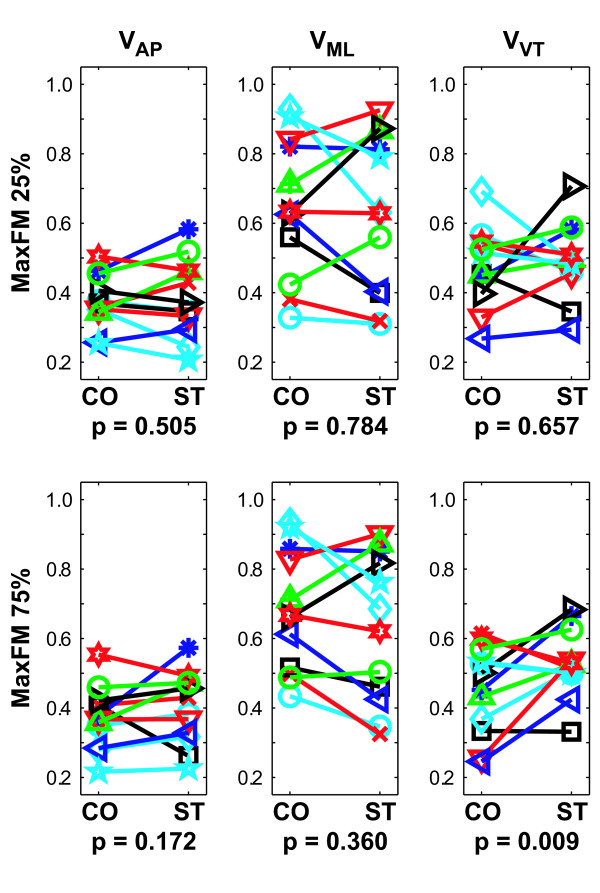
**Orbital stability results.** Magnitudes of maximum Floquet multipliers (MaxFM) for Poincaré sections taken at 25% and 75% of the gait cycle for trunk velocities in the AP, ML, and VT directions. All subjects were orbitally stable (all MaxFM < 1) in all directions, but somewhat less stable (i.e., larger MaxFM) in the ML direction, compared to the AP and VT directions. During the Stroop test, subjects tended to be slightly more stable in the ML direction, but slightly more unstable in the AP and VT directions. This greater instability was statistically significant at the 75% Poincaré section (p = 0.009). Similar results were obtained at the 0%, 50%, and 100% Poincaré sections, but no significant Condition effects (0.231 < p < 0.996) were found. There were no statistically significant Subject × Condition interaction effects for any of the comparisons (0.07 < p < 0.85).

Overall, the Stroop test led to either no changes or slight *in*creases in orbital instability of walking patterns. All subjects exhibited orbitally stable walking kinematics (i.e., Max FM < 1) for all walking trials (Fig. 4), consistent with previous findings [32,42]. In contrast to the local stability findings, Max FM values were, on average, slightly larger (i.e., more *un*stable) for the ST walking condition than the CO walking condition for movements in the AP and VT directions, but slightly smaller (i.e., more stable) for ML movements. None of these differences, however, were statistically significant (0.17 < p < 0.90). At 75% of the gait cycle, subjects did exhibit significantly greater (i.e., more unstable) Max FM values during the Stroop test for vertical movements (p = 0.009; Fig. 4). While none of the Subject × Condition interaction effects were statistically significant (0.07 < p < 0.85), differences between subjects were again evident in the data (Fig. 4).

**Figure 5:**
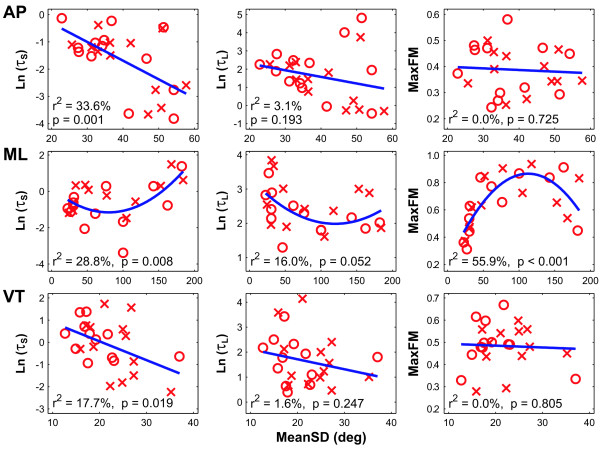
**Regressions between measures of variability (MeanSD) and short-term local divergence time constants (*τ*_*S*_; left column), long-term local divergence time constants (*τ*_*L*_; middle column), and magnitudes of maximum Floquet multipliers (MaxFM; right column) for movements in the AP (top row), ML (middle row), and VT (bottom row) directions.** Each subplot show the average value for each subject for both Stroop ('O') and Control ('X') walking trials. Linear regressions were performed for AP and VT movements, while quadratic regressions were performed for ML movements. Adjusted r^2 ^values and p-values for each regression are shown in each sub-plot. Since each regression contained two data points from each subject, these p-values do not indicate "statistical significance" in the strict sense, but instead indicate only the general quantitative strengths of these relationships.

For AP and VT movements (Fig. 5, top and bottom rows), differences in variability predicted differences in short-term local instability (*τ*_*S*_), but did not predict differences in either long-term local instability (*τ*_*L*_) or orbital instability (MaxFM). For ML movements (Fig. 5, middle row), all three stability measures exhibited quadratic relationships with variability, with trials exhibiting intermediate amounts of variability showing greater instability, while trials exhibiting lesser or greater variability were more stable. We note that since each regression contained dependent data (i.e., 2 data points from each subject), the p-values obtained cannot indicate "statistical significance" in the strict sense. The p-values and r^2 ^values in Fig. 5 instead indicate only the general quantitative strengths of these relationships. Thus, measures of variability and dynamic stability reflected different properties of walking dynamics, consistent with previous findings [9,31].

## Discussion

People often perform secondary attention-demanding cognitive tasks while walking. The apparent relationships between increased fall-risk when performing attention demanding tasks while walking [11-17] and between step kinematic variability and fall risk [19,20,22] suggest that attention demanding tasks might increase step kinematic variability. However, Grabiner and Troy [25] found that young healthy subjects performing a concurrent Stroop task [26] while walking on a motorized treadmill actually exhibited *decreased *step width variability. The relationship between step width and risk of falls remains an issue of debate [28-30] and measures of kinematic variability are not well correlated with measures of dynamic stability that directly quantify the sensitivity of walking kinematics to small perturbations [9,31,32]. Therefore, the present study was conducted to determine if performing the concurrent Stroop task also affected the dynamic stability of walking in the same experiments described in Grabiner and Troy [25].

The present analyses demonstrate that these subjects also exhibited decreased variability of trunk movements in all three principle directions while performing the concurrent Stroop test (Fig. 2). These findings support the decreased step width variability results reported by Grabiner and Troy and demonstrate that this decreased variability was not restricted to leg movements, but also affected trunk movements. Decreasing the variability of trunk (and thereby head) movements during the Stroop test would help subjects stabilize their gaze on the words being projected on the wall [45]. The local and orbital dynamic stability results, however, were mixed. While subjects exhibited somewhat more locally stable movements in the AP direction while performing the Stroop test ('*τ*_*L*_'; Fig. 3C), most comparisons showed minimal differences that were not statistically significant (Fig. 3). Furthermore, subjects exhibited either no significant differences in orbital stability, or slightly greater orbital *in*stability, while performing the Stroop task (Fig. 4). The lack of main effects differences for these measures was likely due at least in part to the fact that different subjects responded differently to the Stroop task, as indicated by the significant interaction effects. Therefore, the decreased variability associated with performing the concurrent Stroop task did *not *translate to greater dynamic stability in these young healthy subjects.

Although subjects did not improve their dynamic stability while performing the Stroop test and walking, they also did not become obviously more *un*stable either. It is likely that these young healthy subjects altered their gait patterns to adapt to the Stroop task, as originally suggested by Grabiner and Troy [25]. However, the present findings demonstrate that they did not *over*-compensate, but were instead able to maintain approximately the same levels of dynamic stability. Another, albeit not mutually exclusive, possibility is that the Stroop test itself imposed constraints for head orientation that were not present in the control task [45]. Thus, the Stroop task may not have been challenging enough to elicit more significant deterioration of dynamic stability during walking. We believe it is likely that we would observe more pronounced effects of concurrent cognitive tasks on the dynamic stability of walking if we examined more impaired (e.g., elderly) populations with more limited capacity to adapt to the task and/or if we required subjects to perform more complex cognitive tasks, such as more complex Stroop test [46,47], or possibly solving arithmetic problems [15,45]. Performing mental arithmetic in particular would likely cause subjects to reorient their visual attention away from external visual landmarks to internal images of the calculation [45], thereby disrupting the otherwise very strong reliance on visual information for the control of walking [48].

One possible limitation of the present study was that subjects walked on a motorized treadmill. Treadmill walking can reduce the natural variability [31,49] and enhance the local stability [31] and, to a lesser extent, the orbital stability [42] of locomotor kinematics. This may be because walking speed is strictly enforced on the treadmill, allowing subjects fewer options for altering their gait speed and/or walking kinematics. The present study needed to be conducted on a motorized treadmill so that walking speeds could be controlled experimentally and to provide the Stroop test intervention. Because each subject walked at the same speed under both conditions, this ensured that comparisons of the variability and dynamic stability between the two walking tasks would remain valid and would not be confounded by subjects changing their gait speed.

None of the subjects tested in this study fell, or even stumbled, during these experiments. As such, the present study was limited to experimentally quantifying how these subjects responded to those small perturbations that occur naturally during normal walking [10,32]. Therefore, these results may or may not extend to *global *stability [39], where the response of the system to much larger perturbations, like tripping or slipping (*e.g*., [50,51]), would be assessed. Clearly, there is a limit to the magnitude of perturbations that humans can accommodate and we do not know how much inherent local or orbital instability humans can tolerate while remaining globally stable. Previous studies showing that obstacle avoidance is also impaired while walking and performing concurrent cognitive tasks [16,17] suggest that global stability is likely also impaired during dual-tasking situations. The present findings, along with our previous work [9,10,35], suggest that the underlying mechanisms responsible for governing local and/or orbital dynamic stability in human locomotion are likely related in some way to those governing global stability. One important line of future research will be to determine if subtle changes in the dynamic stability properties quantified here can also be used to predict the resilience of humans to much larger perturbations.

## Competing interests

The authors declare that they have no competing interests.

## Authors' contributions

MDG and JBD conceived the study. MDG and KLT conducted the experiments and collected the data. JBD evaluated the data and results and was responsible for the initial drafting of the manuscript. RTR wrote/modified software necessary for the analysis and was involved in drafting and revising the manuscript. All authors read and approved the final manuscript.
